# Preparation and Therapeutic Evaluation of Engineered Semaglutide and Statin–Lipid Conjugate-Based Nanoparticle

**DOI:** 10.3390/pharmaceutics17040480

**Published:** 2025-04-07

**Authors:** Kyeong-Ju Lee, Seong-Bin Yang, Jae-Hyeon Lee, Bison Seo, Hyung-Sik Won, Jooho Park

**Affiliations:** BK21 Program, Department of Applied Life Science, Konkuk University, Chungju 27478, Republic of Korea; lkj6709@gmail.com (K.-J.L.);

**Keywords:** nanomedicine, self-assembly, semaglutide, nanoparticle, lipid conjugate

## Abstract

**Background**: Fatty liver disease and obesity are among the most prevalent health conditions in modern society and have recently garnered significant attention. Semaglutide, a well-known anti-obesity drug, has been widely used for diabetes and obesity treatment; however, nanotherapeutics utilizing semaglutide have not yet been developed. **Methods**: A novel statin–lipid conjugate was synthesized using rosuvastatin and ursodeoxycholic acid, a liver-protective agent. This conjugate was then formulated with semaglutide through hydrophobic interactions to create a new nanoparticle system. The physicochemical properties of the nanoparticles were analyzed, and their therapeutic efficacy was evaluated in a high-fat diet (HFD)-induced animal model. **Results**: The statin–lipid conjugate was successfully synthesized, forming novel nanoparticles with semaglutide in an aqueous solution. These nanoparticles exhibited distinct properties compared to conventional semaglutide formulations. In animal experiments, the treatment group demonstrated a 30.24% reduction in body weight and a 46.80% improvement in liver function markers compared to the control group. **Conclusions**: This study introduces a novel semaglutide-based nanoparticle (SRLC NP) system that overcomes key limitations of conventional semaglutide therapy by providing enhanced bioavailability, extended circulation time, and improved cellular uptake. These findings highlight the potential of SRLC NPs as a clinically translatable nanotherapeutic approach for more effective, sustained, and patient-friendly obesity and fatty liver disease treatment.

## 1. Introduction

Obesity and non-alcoholic fatty liver disease (NAFLD) have emerged as critical health concerns in modern society, closely linked to diabetes, cardiovascular diseases, and metabolic syndrome [1,2,3,4]. As one of the most rapidly increasing metabolic disorders worldwide, there is a continuous demand for the development of effective therapeutic agents and treatment strategies [5,6]. Among currently developed pharmaceuticals, semaglutide, a glucagon-like peptide-1 (GLP-1) receptor agonist, has demonstrated significant efficacy in clinical settings for glycemic control and weight reduction, playing a crucial role in the development of therapeutics for diabetes and obesity [7,8,9,10]. However, conventional semaglutide formulations face key challenges, including low oral bioavailability (~1%), rapid enzymatic degradation, and the necessity for frequent subcutaneous injections, leading to suboptimal patient adherence [11,12]. Additionally, semaglutide has poor lipophilicity, limiting its cellular uptake and controlled release potential. To overcome these challenges, nanoparticle-based formulations offer a promising strategy by enhancing drug stability, prolonging circulation time, and facilitating targeted delivery to metabolic tissues. Our novel semaglutide-based nanoparticle system incorporates a rosuvastatin–lipid conjugate (RLC), which not only enhances drug encapsulation and self-assembly but also provides complementary therapeutic benefits in obesity and fatty liver disease. These advancements could significantly improve the clinical applicability of semaglutide while reducing dosing frequency and enhancing therapeutic outcomes. We have elaborated on the key challenges of conventional semaglutide formulations, including low oral bioavailability, enzymatic degradation, and frequent subcutaneous administration requirements [7,9,13].

Nanotechnology-based drug delivery systems have gained significant attention as a promising new approach for optimizing drug stability and efficacy in obesity treatment [14,15,16,17]. The development of semaglutide derivatives and nanomaterial-based drug delivery systems may help overcome several drawbacks associated with existing GLP-1 receptor agonists in treating obesity and NAFLD [16,18,19,20,21]. Nanotechnology can prevent drug degradation, enhance drug stability for long-term storage, and improve drug efficacy [22,23,24,25]. Despite growing interest in nanoparticle formulations of semaglutide, no clinically available semaglutide-based nanoparticles have been developed so far. The future development of semaglutide derivatives and related nanomaterials is expected to introduce a novel therapeutic paradigm in the treatment of diabetes and obesity [26,27].

In our previous studies, we developed various lipid-based bioconjugate and lipid derivatives for treating obesity and liver diseases. Among these, we synthesized statin and lipid conjugates with bile acid moieties, enabling not only the lipid-lowering effects of statins but also additional therapeutic functions [28,29]. The synthesized rosuvastatin and lipid conjugate (RLC) exhibited promising efficacy in animal models of obesity and NAFLD with minimal toxicity. RLC disrupted bile acid metabolism, increasing cholesterol consumption and promoting fat utilization [30], and, additionally, effectively functioned as a statin, successfully inhibiting cholesterol synthesis [31,32,33]. Its effects could suggest the potential for synergistic interactions with semaglutide, and the development of semaglutide-based conjugates could further enhance therapeutic outcomes [34].

**Figure 1:**
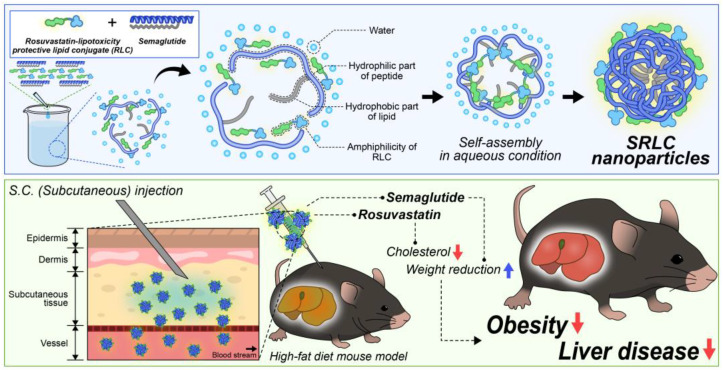
Schematic representation of self-assembled SRLC nanoparticles in aqueous conditions due to their amphiphilic properties. SRLC NPs exert anti-obesity and fatty liver improvement effects through the synergistic action of rosuvastatin and semaglutide following subcutaneous administration.

In this study, we newly developed a novel nanoparticle formulation utilizing semaglutide and previously synthesized statin–fatty acid derivatives (RLC). The nanoparticles were self-assembled based on hydrophobic interactions and van der Waals forces between the fatty acid moieties of statins and semaglutide (Figure 1). Lipid-based conjugates facilitated nanoparticle formation by providing amphiphilic properties that promoted self-assembly and enhanced structural stability [35,36]. The resulting semaglutide–rosuvastatin–lipid conjugate (SRLC) nanoparticles were well defined and exhibited high stability in aqueous solutions. Upon administration in high-fat diet (HFD)-induced obese animal models, these nanoparticles demonstrated significant anti-obesity effects, including substantial weight reduction, and maintained some liver injury markers, thereby providing hepatoprotective benefits. The successful development of these nanoparticles highlights their potential in advancing nanomaterial-based therapeutics for obesity treatment, paving the way for future applications in metabolic disease management.

## 2. Materials and Methods

### 2.1. Materials

Acetonitrile (ACN), antibiotic antimycotic solution (100×), anhydrous dimethyl sulfoxide (DMSO), chloroform, Dulbecco’s modified Eagle’s medium (DMEM), Dulbecco’s phosphate-buffered saline (DPBS), dimethyl sulfoxide-d_6_, ethanol, 1-ethyl-3-(3-dimethyl aminopropyl) carbodiimide (EDC), ethylenediamine (EDA), ether, methanol, N-hydroxysuccinimide (NHS), 2-methyl-2-butanol, phosphate-buffered saline (PBS), rhodamine B isothiocyanate (RITC), semaglutide, trifluoroacetic acid (TFA), 2,2,2-tribromoethanol (TBE), and ursodeoxycholic acid (UDCA) were purchased from Sigma Aldrich (St. Louis, MO, USA). Fetal bovine serum (FBS) was obtained from Gibco (Milford, MA, USA). Rosuvastatin calcium (RO) was obtained from Aladdin Co., Ltd. (Shanghai, China). Hydrochloric acid (35.0–37.0%) was purchased from SAMCHUN (Seoul, Republic of Korea). The EZ-cytox kit was from DoGenBio (Seoul, Republic of Korea). D(+)-glucose was purchased from Duksan (Ansan, Republic of Korea).

### 2.2. Preparation and Characterization of Rosuvastatin–Lipid Conjugate (RLC)

The synthesis of the rosuvastatin–lipid conjugate (RLC) was performed through a three-step reaction process. In the first step, 50 mg of rosuvastatin (RO) was dissolved in anhydrous DMSO along with 77.5 mg of 1-ethyl-3-(3-dimethyl aminopropyl) carbodiimide (EDC) and 34.5 mg of *N*-hydroxysuccinimide (NHS). The reaction mixture was continuously stirred at ambient temperature for a duration of four hours to facilitate the synthesis of RO-NHS. Simultaneously, another anhydrous DMSO solution dissolved 100 mg of ursodeoxycholic acid (UDCA), EDC (197.7 mg), and NHS (87.9 mg) was stirred under the same conditions to synthesize UDCA-NHS. In the second step, 306.2 mg of ethylenediamine (EDA) was added to the UDCA-NHS solution, followed by an additional 10 min of stirring to produce UDCA-EDA. Each reaction mixture was precipitated in 30 mL of distilled water (DW) for purification and subsequently subjected to centrifugation at 4000 rpm and 4 °C for 5 min. This purification process was repeated three times to ensure thorough removal of by-products. In the final step, RO-NHS (5 mg) and UDCA-EDA (18.8 mg) were dissolved in 3 mL of anhydrous DMSO and stirred overnight at room temperature to complete the synthesis of RLC. The reaction mixture was then precipitated in 30 mL of distilled water, followed by purification through centrifugation at 4000 rpm and 4 °C for 4 min, repeated three times. The purified product was lyophilized to obtain white powder. For additional purification, the product was precipitated in a diethyl ether–acetonitrile (9:1) solution, sonicated for 5 min, and subjected to centrifugation at 2500 rpm and 4 °C for 4 min. This process was repeated five times to ensure high purity. Finally, the purified RLC was obtained as a white lyophilized powder.

Each reaction was monitored using thin-layer chromatography (TLC) with a mobile phase composed of methanol and chloroform at a 1:3 volume ratio. The purity of the synthesized compounds was further validated using reversed-phase high-performance liquid chromatography (RP-HPLC) (Agilent 1200 series, Agilent Technologies, Santa Clara, CA, USA) equipped with a ZORBAX RR Eclipse Plus C18 column (4.6 mm × 150 mm, 3.5 μm). A gradient elution method was employed, where the ratio of solvent A (acetonitrile containing 0.1% TFA) to solvent B (distilled water containing 0.1% TFA) varied from 10:90 to 90:10 over 40 min. The flow rate was set to 1 mL/min, and the elution profile was tracked with a UV–vis detector at 234 nm.

### 2.3. Preparation and Stability Optimization of SRLC NPs

To determine the optimal formulation and final composition of the semaglutide–RLC nanoparticles (SRLC NPs), a precipitation evaluation experiment was conducted using various mass ratios (1:10, 1:20, 1:30, *w*/*w*) of semaglutide to RLC. Based on stability and particle size analysis, the 1:10 ratio was selected as the optimal formulation, and this ratio was maintained in the final product composition to ensure consistency in therapeutic efficacy and physicochemical properties. The resulting mixture was used immediately without undergoing a separate freeze-drying process. For nanoparticle preparation, semaglutide (0.5 mg) was dissolved in 1 mL of DW, while RLC (2 mg) was dissolved in 1 mL of DMSO. Based on a fixed semaglutide concentration of 0.1 mg, different amounts of RLC were added to prepare solutions with varying semaglutide–RLC (1:10, 1:20, 1:30) mass ratio in 1 mL of DW containing 5% DMSO. The stability of each formulation was assessed by measuring particle size at multiple time points (0, 0.5, 1, 2, 3, 4, and 5 days) using dynamic light scattering (DLS; Zetasizer Nano, Malvern Instruments, Worcestershire, UK). The zeta potential of both RLC alone and the semaglutide–RLC (1:10) formulation was also measured to evaluate the surface charge and colloidal stability of the nanoparticles. Additionally, in vivo stability was evaluated by preparing 1 mL of Dulbecco’s phosphate-buffered saline (DPBS) containing 10% fetal bovine serum (FBS) and 5% DMSO, followed by particle size measurements at different time points (0, 0.5, 1, and 2 days). The particle formation and size distribution of SRLC NPs were analyzed using DLS to assess their stability under physiologically relevant conditions.

### 2.4. Nanoparticle Analysis Using Electron Microscopy

Transmission electron microscopy (TEM) and scanning electron microscopy (SEM) were conducted to examine the morphology and structural characteristics of SRLC NPs. For TEM, samples were deposited onto a formvar/carbon-coated nickel (Ni) grid, stained with 5% uranyl acetate for 3 min, washed, and air-dried before imaging. SEM analysis was performed using a MERLIN (Carl-Zeiss, Oberkochen, Germany) scanning electron microscope in high-resolution mode with a secondary electron (SE) detector. Samples were prepared by mounting onto a silicon wafer, sequentially dehydrating through a graded ethanol series, and coating with a gold layer to enhance conductivity. The imaging parameters were configured with an accelerating voltage of 10 kV, a working distance of 10 mm, and a probe current of 160 pA, enabling detailed visualization of the morphology and surface features of SRLC NPs.

### 2.5. In Silico Computer Simulation of Nanoformulation

The chemical structure of RLC was constructed using ChemDraw Professional 20.1.1.125 (PerkinElmer Inc., Los Angeles, CA, USA), while the semaglutide structure was obtained from the Protein Data Bank (PDB ID: 4ZGM). Both structures were parameterized using the Chemistry at HARvard Molecular Mechanics (CHARMm) force field for molecular dynamics simulations. The simulations were performed in Discovery Studio 2022 software (BIOVIA, San Diego, CA, USA) using the Standard Dynamics Cascade protocol under a distance-dependent dielectric solvent model within a constant isobaric–isothermal (NPT) ensemble. During the 200 ps production phase, trajectory analysis was conducted to evaluate hydrogen bond formation and molecular interactions. The optimized molecular conformations were visualized using Discovery Studio.

### 2.6. Cytotoxicity Assay of SRLC NPs in Fibroblast Cell

The cytotoxicity of the RLC and SRLC nanoparticles was evaluated using fibroblast cells obtained from the Korean Cell Line Bank (Seoul, Republic of Korea). The fibroblast cells were cultured in high-glucose Dulbecco’s modified Eagle’s medium (DMEM) supplemented with 10% (*v*/*v*) fetal bovine serum (FBS) and 1% antibiotic–antimycotic solution. The cells were then seeded into a 96-well culture plate at a density of 1.0 × 10^4^ cells per well and incubated for 1 h at 37 °C in a humidified atmosphere containing 5% CO_2_ to allow stabilization. Following stabilization, the cells were treated with RLC and SRLC nanoparticles at concentrations ranging from 0.1 to 100 μM for 24 h. Cell viability (*n* = 5) was evaluated using the EZ-Cytox cell viability assay kit, with absorbance readings taken at 450 nm and 600 nm using a microplate reader (SPECTROstar Nano, BMG LABTECH, Ortenberg, Germany). The viability of the treated cells was determined by comparing the UV absorbance values of the samples to those of the untreated control group.

### 2.7. Preparation of RITC-Labeled Semaglutide

To evaluate cellular uptake, RITC-labeled semaglutide was synthesized by dissolving 10 mg of semaglutide in 2 mL of anhydrous dimethylformamide (DMF) under continuous stirring. A separate solution of RITC (1 mg in 1 mL DMF) was prepared and gradually added to the semaglutide solution. The reaction mixture was stirred at 600 rpm at room temperature overnight to facilitate labeling. After incubation, 20 mL of distilled water was introduced into the reaction system while applying ultrasonic dispersion to ensure homogeneity. The resulting solution was purified through centrifugation (4000 rpm, 5 min, 4 °C), a process repeated three times to remove unreacted components. Finally, the purified labeled semaglutide was lyophilized, and successful RITC conjugation was validated using thin-layer chromatography (TLC) with a mobile phase composed of methanol and chloroform (1:4, *v*/*v*).

### 2.8. In Vivo Pharmacokinetic (PK) Analysis

The male SD rats (6-week-old) were housed for one week in an environment with controlled temperature and humidity under a 12 h light–dark cycle. RITC-labeled semaglutide (1 mg) and RLC (10 mg) were dissolved in distilled water to prepare SRLC NPs. The nanoparticles were administered subcutaneously at a semaglutide concentration of 1 mg/kg. After administration, blood was collected from the jugular vein at each time point (0, 0.5, 1, 3, 6, 12, 24, 48, 72, 96, 120, 144, and 168 h) and allowed to clot at room temperature for 30 min. It was then centrifuged at 4500 G for 15 min at 4 °C to collect the serum. The collected serum was mixed with DMSO at a 1:4 volume ratio for fluorescence measurement. Fluorescence intensity was measured using a multi-mode microplate reader (SpectraMax M2/SpectraMax L, Molecular Devices, San Jose, CA, USA) at an excitation/emission wavelength of λ_Ex_ 555/λ_Em_ 595.

### 2.9. High-Fat Diet (HFD) Animal Experiment

All animal experiments were conducted under the standard regulations of the Institutional Animal Care and Use Committee (IACUC) at Konkuk University (ref. no. KU22078-1). The animals were housed in a controlled environment with regulated temperature and humidity, maintained under a 12 h light–dark cycle. Obesity was induced in 5-week-old male C57BL/6 mice through dietary control for 69 days. A total of 25 mice were used and divided into five experimental groups: negative control (regular diet, RD), positive control (high-fat diet, HFD), RO (0.5 mg/kg), RLC (1 mg/kg), and SRLC NPs (1.1 mg/kg). The RO dose was set at half that of RLC due to its molecular weight being approximately twice that of RLC, ensuring equivalent molar exposure. Additionally, the SRLC NP dose was adjusted to 1.1 mg/kg as it comprised 1 mg of RLC and 0.1 mg of semaglutide, allowing for the accurate comparison of therapeutic effects. While the RD group received a normal diet, the other groups were fed a high-fat diet (HFD) consisting of 60 kcal% fat (D12492, Research Diets, New Brunswick, NJ, USA). The body weights of the mice were measured weekly. Starting from day 28 of the dietary intervention, each group received their respective substances via subcutaneous injection every three days. Five days before the end of the experiment, an oral glucose tolerance test (OGTT) was conducted to assess glucose sensitivity. After 19 h of fasting, all experimental groups were administered a glucose solution orally (2 g/kg). Blood glucose levels were then measured at time intervals of 0, 15, 30, 60, 90, 120, and 180 min (*n* = 3). Blood glucose levels were assessed using a glucometer (G CARE, GC Medical Science, Yongin, Republic of Korea). On the final day of the experiment, after 14 h of fasting, the mice were anesthetized via intraperitoneal administration of 2,2,2-tribromoethanol (TBE), and blood samples were collected. Hepatotoxicity and lipid profiling analyses were conducted using the collected blood samples, while liver tissues were subjected to hematoxylin and eosin (H&E) and oil red O (ORO) staining for further evaluation. All analyses were performed by KP&T Technology (Cheongju, Republic of Korea).

### 2.10. Statistical Analysis

The results were expressed as mean ± standard deviation (S.D.), with error bars indicating the S.D. of the mean from independent samples. Statistical differences between groups were evaluated using a one-way ANOVA followed by post hoc analysis. All statistical analyses were conducted using GraphPad Prism 9.0 (GraphPad Software Inc., San Diego, CA, USA) or SigmaPlot 12.0 (Systat Software Inc., Palo Alto, CA, USA). A *p*-value of less than 0.05, 0.01, or 0.001 was considered statistically significant (* *p* < 0.05, ** *p* < 0.01, and *** *p* < 0.001).

## 3. Results and Discussion

### 3.1. Synthesis of RLC

**Figure 2:**
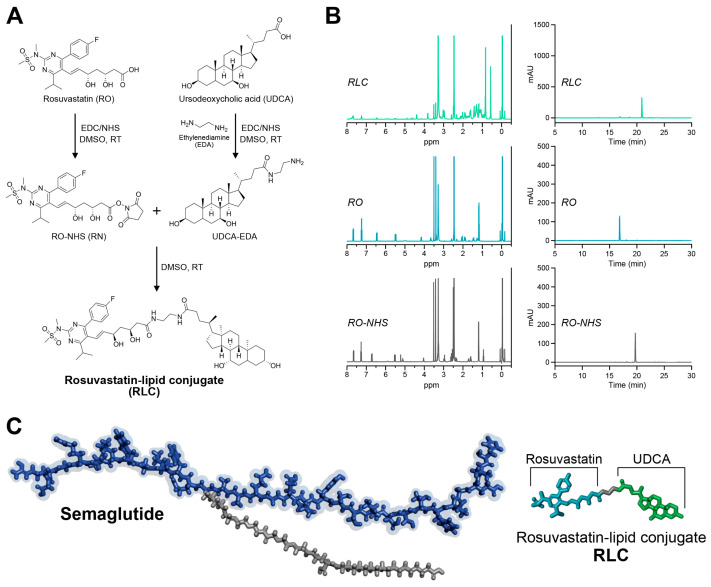
Characterization of rosuvastatin–lipid conjugate (RLC). (**A**) Synthesis scheme of RLC. (**B**) The ^1^H-NMR spectrum analysis and RP-HPLC results of RLC. (**C**) 3D molecular structure of semaglutide and RLC molecules.

The rosuvastatin–lipid conjugate (RLC) was synthesized through a three-step reaction using dimethyl sulfoxide (DMSO) as a solvent and an EDC/NHS (1-ethyl-3-(3-dimethyl aminopropyl) carbodiimide/*N*-hydroxysuccinimide) coupling reaction (Figure 2A). In this process, the carboxyl groups of rosuvastatin (RO) and ursodeoxycholic acid (UDCA) were chemically conjugated to the amino groups at both ends of ethylenediamine (EDA). The successful synthesis and purity of RLC were validated using one-dimensional proton nuclear magnetic resonance (^1^H-NMR) spectroscopy and reversed-phase high-performance liquid chromatography (RP-HPLC). Figure 2B shows the updated results, with clear labeling for RO, RO-NHS, UDCA-EDA, and the final RLC product. The ^1^H-NMR spectra of RLC exhibited characteristic shifts confirming successful conjugation. The RO spectrum displayed peaks at δ 6.8–7.2 ppm, corresponding to aromatic protons, and a peak at δ 1.2–1.4 ppm, indicative of aliphatic chains. After the formation of RO-NHS, a new peak at δ 2.8–3.0 ppm appeared, confirming the activation of the carboxyl group. The UDCA-EDA spectrum revealed amide bond formation, with an upfield shift at δ 7.9–8.1 ppm, suggesting successful EDA incorporation. In the final RLC spectrum, the disappearance of the NHS-related peak and the presence of a broad peak around δ 8.0 ppm confirmed amide bond formation between RO and UDCA-EDA. These spectral changes validated the structural modifications and successful conjugation, distinguishing RLC from its precursor compounds (Figure 2B). Additionally, The RP-HPLC analysis showed a distinct retention time (RT) for RLC, differentiating it from RO and RO-NHS (Figure 2B). The synthesized hydrophobic statin molecule, RLC, was expected to successfully interact hydrophobically with semaglutide, which possessed a hydrophobic fatty acid chain in its molecular structure (Figure 2C). This structural modification was expected to enhance the molecular stability and bioavailability of the conjugated complex, potentially improving its properties.

### 3.2. In Silico Molecular Dynamics (MD) Simulation of SRLC NPs

**Figure 3:**
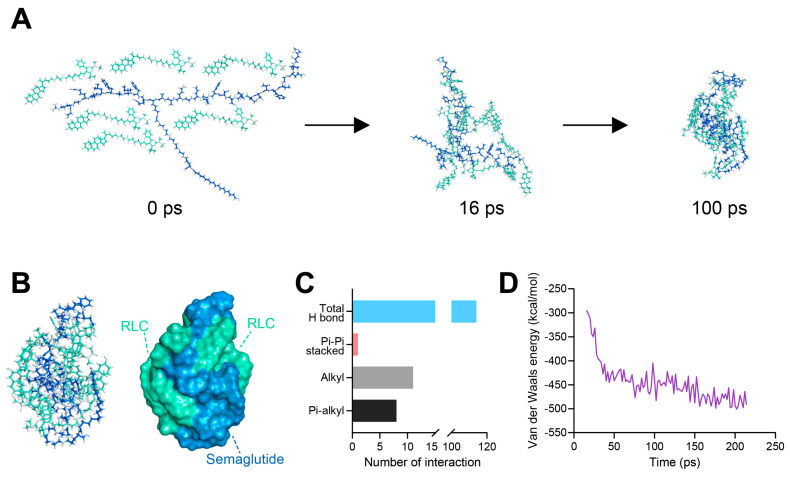
In silico analysis of self-assembled SRLC NPs. (**A**) Molecular dynamics (MD) simulations to confirm the self-assembly pattern of SRLC NPs. (**B**) Visualization of interactions within the SRLC NPs, with components shown in distinct colors: green for RLC and blue for the semaglutide molecule. (**C**) The number of interactions calculated in molecular dynamics in the SRLC NPs simulation models. (**D**) Total Van der Waals energy in the MD simulations of SRLC NPs.

To validate the nanoformulation of SRLC NPs in silico, molecular dynamics (MD) simulations were performed for 0 to 200 ps using BIOVIA Discovery Studio software. Under the CHARMm (Chemistry at Harvard Macromolecular Mechanics) force field, SRLC NPs spontaneously self-assembled into nanoparticles over time (Figure 3A), with the RLC and semaglutide groups exhibiting complex intermolecular interactions, thereby maintaining a stable structure (Figure 3B). The simulated SRLC NPs displayed various molecular interactions, including hydrogen bonds (H-bonds), π–π stacking, alkyl interactions, and π-alkyl interactions, indicating an increased complexity in the intermolecular interactions between the RLC and semaglutide molecules (Figure 3C). Furthermore, the Van der Waals energy decreased from −295.41 kcal/mol at 16 ps to −493.57 kcal/mol at 200 ps, indicating a transition to a more thermodynamically stable state (Figure 3D). Furthermore, the stable self-assembly and enhanced intermolecular interactions suggested that SPLC NPs could achieve a thermodynamically favorable nanoformulation, reinforcing their potential for efficient drug delivery.

### 3.3. Characterization of SRLC NPs

**Figure 4:**
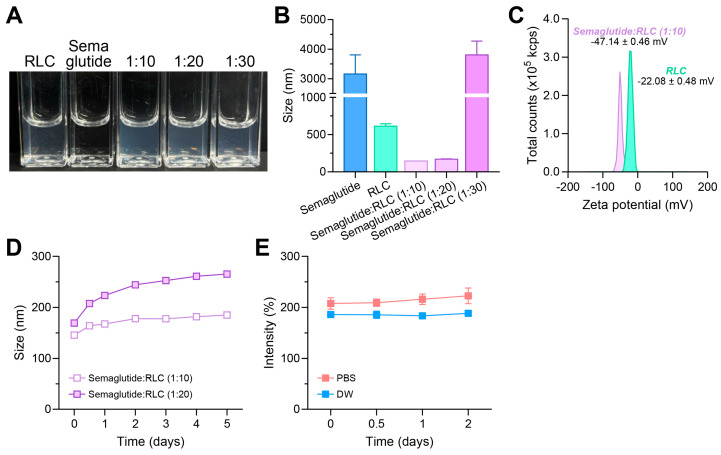
Nano-characterization and stability analysis of SRLC NPs. (**A**) Particle formation under aqueous conditions and (**B**) average size of particles (*n* = 3) in various mass ratios. (**C**) Zeta potential of RLC and SRLC NPs. (**D**) Size stability of SRLC NPs over 5 days. (**E**) Size stability test of SRLC NPs under physiological conditions.

To determine the most stable formulation ratio between RLC and semaglutide, the particle size in an aqueous solution was measured using dynamic light scattering (DLS). Various mass ratios (semaglutide–RLC = 1:10, 1:20, 1:30) were prepared in 1 mL of distilled water (DW) containing 5% DMSO, with the concentration of semaglutide fixed at 0.1 mg (Figure 4A). The particle sizes of each component were measured as follows: semaglutide at 0.1 mg/mL, 3159.67 ± 526.64 nm; RLC at 1 mg/mL, 613.30 ± 26.86 nm; 1:10 mass ratio, 145.80 ± 0.99 nm; 1:20 mass ratio, 170.40 ± 3.11 nm; and 1:30 mass ratio, 3804.67 ± 382.20 nm (Figure 4B). To further assess the colloidal stability of the formulations, zeta potential measurements were conducted (Figure 4C). The RLC alone exhibited a zeta potential of −22.08 ± 0.48 mV, whereas the semaglutide–RLC (1:10) formulation showed a significantly lower zeta potential of −47.14 ± 0.46 mV. This notable decrease in surface charge suggested enhanced electrostatic repulsion, which contributed to the improved colloidal stability of SRLC NPs and reduced the likelihood of aggregation. Since only the 1:10 and 1:20 ratios formed nanoparticles, further stability evaluations were conducted for these formulations over 5 days (Figure 4D). The results showed that the particle size remained stable at 184.93 ± 0.98 nm for the 1:10 ratio and 265.20 ± 3.41 nm for the 1:20 ratio, indicating that the 1:10 ratio formed more stable nanoparticles. This optimized formulation was designated as semaglutide–RLC nanoparticles (SRLC NPs). Furthermore, the stability of SRLC NPs was monitored under various physiological conditions for two days (Figure 4E). In DPBS containing 10% FBS and 5% DMSO, the particle size was 222.60 ± 12.54 nm, while in DW containing 10% FBS and 5% DMSO, it remained stable at 188.27 ± 1.08 nm. These results demonstrated that SRLC NPs maintained structural stability without aggregation, forming a stable colloidal dispersion even in serum-containing environments. This characteristic is particularly beneficial as it suggests that SRLC NPs are less susceptible to enzymatic degradation and premature clearance, thereby remaining in circulation for an extended duration. Such prolonged systemic exposure may enhance bioavailability and sustain therapeutic efficacy, contributing to their potential clinical benefits in treating obesity and fatty liver disease. The observed stability across different media suggests that the SRLC NPs could resist premature aggregation or degradation, potentially leading to improved bioavailability and prolonged circulation time in vivo.

### 3.4. Morphological Analysis of SRLC NPs

**Figure 5:**
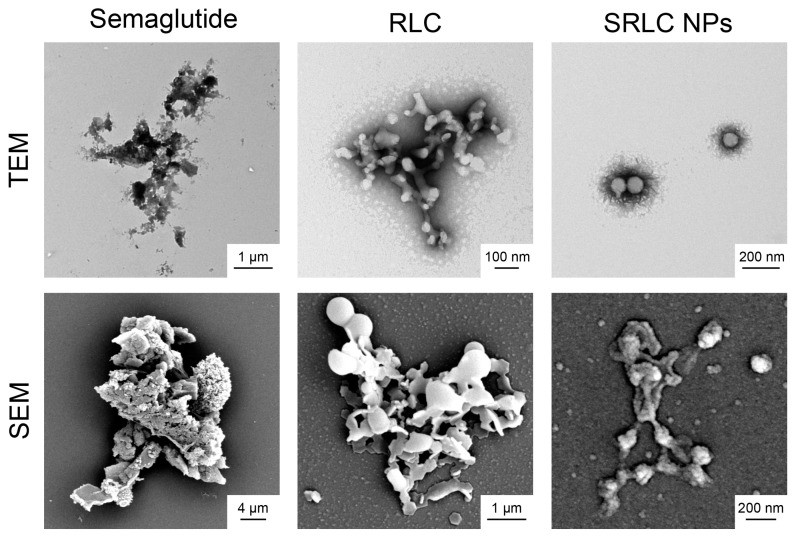
Morphological analysis of SRLC NPs using TEM/SEM images.

The morphology of the SRLC NPs was observed using transmission electron microscopy (TEM) and scanning electron microscopy (SEM), providing a detailed analysis of the nanoparticles’ morphological characteristics. The analysis showed that semaglutide and RLC formed irregular shapes and micron-sized particles (Figure 5). However, SRLC NPs were observed to form well-dispersed, spherical nanostructures, suggesting successful nanoformulation through self-assembly. The TEM images confirmed the presence of a distinct core–shell structure, which may indicate the encapsulation or strong intermolecular interaction between RLC and semaglutide. The SEM analysis further supported these findings, showing a significant reduction in particle size and increased uniformity upon nanoformulation. This well-defined nanostructure suggests that SRLC NPs could enhance drug stability and bioavailability, addressing the limitations of peptide-based therapeutics like semaglutide.

### 3.5. Cytotoxicity Assay of SRLC NPs on Fibroblast Cell

**Figure 6:**
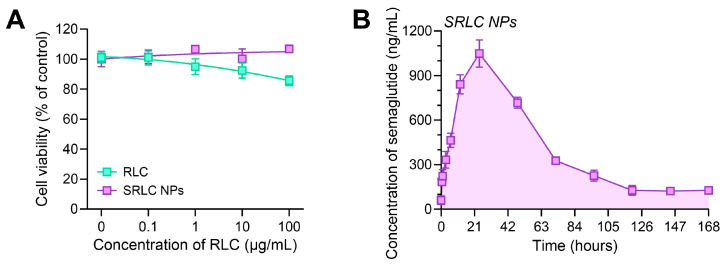
Evaluation of cytotoxicity and pharmacokinetic (PK) Analysis of SRLC NPs. (**A**) Cell viability test of fibroblast cells in the concentration range of 0.1–100 µM RLC and SRLC NPs (*n* = 5). (**B**) Blood concentration profile of SRLC NPs in rats following subcutaneous (S.C.) injection (*n* = 3).

To evaluate the cytotoxic effects of SRLC NPs, a cytotoxicity assay was performed using fibroblast cells at concentrations ranging from 0.1 to 100 µg/mL (Figure 6A). Interestingly, SRLC NPs exhibited reduced cytotoxicity compared to RLC. While RLC showed cytotoxicity at 100 µg/mL with a cell viability of 85.60 ± 2.87%, SRLC NPs displayed no cytotoxicity within this concentration range, with a cell viability of 106.80 ± 2.14%. SPLC NPs maintained cell viability above 100%, suggesting that the nanoformulation not only mitigated toxicity but may have also promoted cellular compatibility. These results suggest that (1) the nanoformulation of SRLC NPs improves the intracellular dispersion of RLC, preventing its accumulation and the formation of locally high toxic concentrations, and (2) the gradual drug release characteristic of the nanoparticles contributes to improved overall safety.

Following the confirmation of biocompatibility through in vitro cytotoxicity assays, a blood distribution analysis was conducted in rats to assess the systemic behavior and bioavailability of SRLC NPs (Figure 6B). Using RITC-labeled semaglutide, the pharmacokinetic analysis (PK) demonstrated that SRLC NPs achieved a peak plasma concentration (Cmax) at approximately 22 h post injection, followed by a rapid decline before reaching a steady state at lower concentrations. This PK profile suggested an initial rapid absorption and systemic distribution, followed by early-phase clearance or tissue uptake. The steady-state phase indicated that a fraction of the encapsulated semaglutide remained in circulation, enabling sustained receptor activation. Such prolonged systemic exposure may contribute to continuous glucose and lipid metabolism regulation, reinforcing the potential therapeutic benefits of SRLC NPs in metabolic disorders.

### 3.6. Anti-Obesity Effect of SRLC NPs in High-Fat Diet (HFD)-Fed Mouse Model

**Figure 7:**
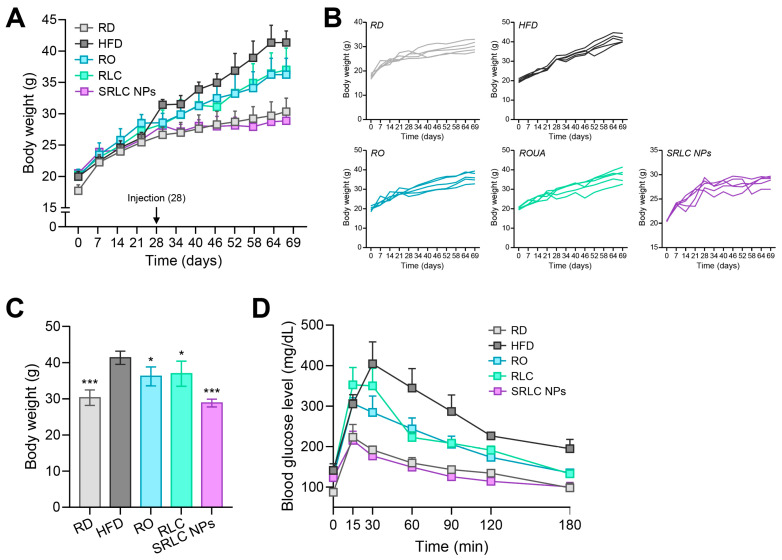
Anti-obesity effect of SRLC NPs in a high-fat diet (HFD)-fed mouse model. (**A**) Body weight changes after 69 days. (**B**) Individual body weight change trend over 69 days and (**C**) body weight on the final day of administration with RO (0.5 mg/kg), RLC (1 mg/kg), and SRLC NPs (1.1 mg/kg) in HFD-fed mouse (*n* = 5). * *p* < 0.05 and *** *p* < 0.001 vs. HFD. (**D**) Oral glucose tolerance test (OGTT) performed on day 64.

To evaluate the anti-obesity effect of SRLC NPs, obesity was induced in mice by feeding them a high-fat diet (HFD) containing 60 kcal% fat (D12492, Research Diets, New Brunswick, NJ, USA). Mice were subcutaneously administered RO (0.5 mg/kg), RLC (1 mg/kg), or SRLC NPs (1.1 mg/kg) every three days (Figure 7A). Compared to the regular-diet (RD) group, the HFD-fed group exhibited a significant increase in body weight. However, SRLC NPs administration effectively suppressed weight gain, leading to a body weight reduction comparable to that of the RD group (Figure 7B). At the end of the experiment, the body weights of the mice were as follows: RD, 30.30 ± 1.92 g; HFD, 41.34 ± 1.64 g; RO, 36.20 ± 2.34 g; and SRLC NPs, 28.84 ± 0.96 g (Figure 7C). Five days before the conclusion of the experiment, an oral glucose tolerance test (OGTT) was performed to evaluate the effects of SRLC NPs on diabetes-related metabolic disorders (Figure 7D). The HFD, RO, and RLC groups exhibited a sharp increase in blood glucose levels immediately after administration, followed by a gradual decline. In contrast, the RD and SRLC NPs groups demonstrated a more stable glucose response, characterized by minimal initial spikes and a steady decrease over time, suggesting improved glucose regulation. These results suggest that SRLC NPs effectively mitigate excessive fat accumulation and impaired glucose regulation. These results suggest that SRLC NPs not only prevent excessive fat accumulation but also improve glucose homeostasis, potentially mitigating obesity-related metabolic disorders. The anti-obesity effects of SRLC NPs may be attributed to their role in regulating lipid metabolism and insulin sensitivity. As nanoparticle formulations can enhance bioavailability, SRLC NPs may exert stronger metabolic benefits than their individual components. Further studies are required to elucidate their precise molecular mechanisms and long-term safety. In conclusion, SRLC NPs effectively reduce weight gain and improve glucose metabolism in HFD-induced obese mice, highlighting their potential as a novel therapeutic strategy for obesity and related metabolic disorders.

### 3.7. Clinical Pathological Evaluation of the Therapeutic Effects of SRLC NPs in High-Fat Diet (HFD)-Fed Mouse Model

**Figure 8:**
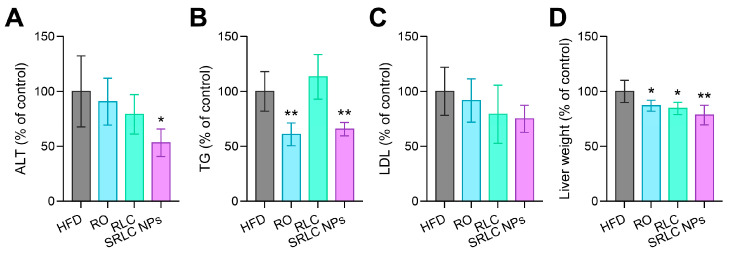
Clinical pathology analysis of SRLC NPs. (**A**) Aminotransferase (ALT), (**B**) triglyceride (TG), (**C**) low-density lipoprotein (LDL), and (**D**) liver weight after treatment with RO (0.5 mg/kg), RLC (1 mg/kg), and SRLC NPs (1.1 mg/kg) in high-fat diet (HFD)-fed mouse (*n* = 5). * *p* < 0.05 and ** *p* < 0.01 vs. HFD.

After the completion of the 69-day experiment, a clinical pathology analysis was performed to assess the metabolic and hepatic effects of SRLC NPs. Despite continuous HFD feeding, the SRLC NPs-treated group exhibited significant reductions in alanine aminotransferase (ALT) levels (Figure 8A), triglyceride (TG) levels (Figure 8B), low-density lipoprotein (LDL) levels (Figure 8C), and liver weight (Figure 8D) compared to the HFD group. These findings suggest that SRLC NPs effectively mitigate obesity-induced hepatic dysfunction and metabolic abnormalities, potentially protecting against liver damage caused by excessive fat accumulation. Furthermore, the improvements in liver enzyme levels and lipid profiles indicate that SRLC NPs may help regulate lipid metabolism and alleviate hepatic damage associated with obesity and metabolic diseases. The ability of SRLC NPs to reduce liver weight and lipid accumulation suggests their potential role in preventing non-alcoholic fatty liver disease (NAFLD) and related metabolic complications. Therefore, SRLC NPs could serve as a promising therapeutic option for improving pathological changes caused by obesity and metabolic disorders, warranting further studies to explore their long-term efficacy and underlying mechanisms.

### 3.8. Histopathological Evaluation of the Therapeutic Effects of SRLC NPs in High-Fat Diet (HFD)-Fed Mouse Model

**Figure 9:**
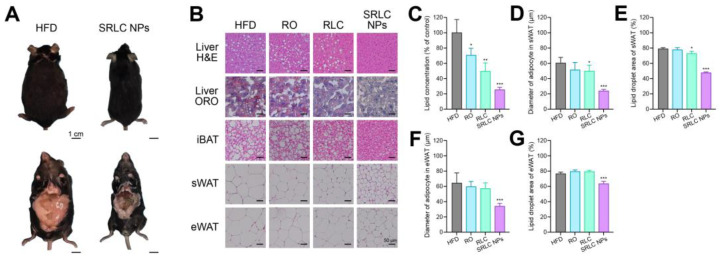
Histopathological analysis of SRLC NPs. (**A**) Representative image of high-fat diet (HFD)-fed and SRLC NP-treated mice. (**B**) Histology image after treatment with RO (0.5 mg/kg), RLC (1 mg/kg), and SRLC NPs (1.1 mg/kg) in HFD-fed mice. (**C**) Quantification of ORO staining (*n* = 4). * *p* < 0.05, ** *p* < 0.01, and *** *p* < 0.001 vs. HFD. (**D**) Diameter of adipocyte in sWAT (*n* = 7). * *p* < 0.05 and *** *p* < 0.001 vs. HFD. (**E**) Area of adipocyte in sWAT (*n* = 4). * *p* < 0.05 and *** *p* < 0.001 vs. HFD. (**F**) Diameter of adipocyte in eWAT (*n* = 7). *** *p* < 0.001 vs. HFD. (**G**) Area of adipocyte in eWAT (*n* = 4). *** *p* < 0.001 vs. HFD.

Representative images of mice from the HFD group and the SRLC NPs-treated group on the final day of the experiment are shown in Figure 9A. To evaluate the pathological changes at the tissue level and the therapeutic effects of SRLC NPs, hematoxylin and eosin (H&E) staining and oil red O (ORO) staining were performed (Figure 9B). These staining analyses were conducted to confirm the reduction in hepatic fat accumulation and assess adipose tissue remodeling following SRLC NPs treatment. H&E and ORO staining revealed abnormal fat droplet accumulation in the HFD, RO, and RLC groups. In contrast, the SRLC NP-treated group exhibited a significant reduction in both lipid staining and fat droplet accumulation in liver tissue, suggesting an improvement in hepatic lipid metabolism. These findings suggest that subcutaneous administration of SRLC NPs has the therapeutic potential to reduce blood lipid levels and ameliorate fatty liver conditions. Additionally, the results of H&E staining showed that subcutaneous white adipose tissue (sWAT) in the SRLC NP-treated group exhibited a more uniform cell size and regular shape, indicating a healthier adipose morphology. Furthermore, the diameters of adipocytes in interscapular brown adipose tissue (iBAT), sWAT, and epididymal white adipose tissue (eWAT) were significantly reduced in the SRLC NP-treated group compared to the HFD group, further supporting its anti-obesity effects. Quantification of ORO staining showed the following values (*n* = 4): HFD, 100.00 ± 15.00%; RO, 70.50 ± 8.02%; RLC, 49.50 ± 9.53%; and SRLC NPs, 25.25 ± 2.95% (Figure 9C). Additionally, significant differences were observed in adipocyte size and area: The diameter of the adipocyte in sWAT (*n* = 7) was as follows: HFD, 60.32 ± 6.95 µm; RO, 51.20 ± 8.99 µm; RLC, 49.79 ± 7.09 µm; and SRLC NPs, 23.75 ± 2.01 µm (Figure 9D). The area of the adipocyte in sWAT (*n* = 4) was as follows: HFD, 78.86 ± 1.38%; RO, 77.51 ± 2.52%; RLC, 72.72 ± 2.75%; and SRLC NPs, 47.45 ± 1.03% (Figure 9E). The diameter of the adipocyte in eWAT (*n* = 7) was the following: HFD, 64.12 ± 12.51 µm; RO, 59.53 ± 6.42 µm; RLC, 57.01 ± 7.04 µm; and SRLC NPs, 33.79 ± 3.62 µm (Figure 9F). The area of the adipocyte in eWAT (*n* = 4) was as follows: HFD, 76.44 ± 1.91%; RO, 79.30 ± 1.91%; RLC, 79.223 ± 1.53%; and SRLC NPs, 63.28 ± 2.94% (Figure 9G). These findings suggest that SRLC NPs effectively mitigate obesity-induced metabolic abnormalities, including excessive fat accumulation and adipose tissue hypertrophy, potentially contributing to overall metabolic improvements.

## 4. Conclusions

In this study, we successfully developed semaglutide–RLC nanoparticles (SRLC NPs) through the optimized formulation of a rosuvastatin–lipid conjugate (RLC) with semaglutide. Based on formulation stability and characterization studies, a semaglutide–RLC ratio of 1:10 (*w*/*w*) was determined to be optimal and was used for the final nanoparticle preparation. SRLC NPs exhibited remarkable physicochemical stability under physiologically relevant conditions and formed well-defined nanoscale structures, as confirmed by multiple analytical techniques. Importantly, the particle size of the SRLC NPs was approximately 200 nm, significantly smaller than that of the individual components, indicating successful self-assembly. Moreover, the SRLC NPs maintained stability without aggregation in bovine serum-containing media, indicating their potential for prolonged systemic circulation and enhanced bioavailability. Their structural integrity and uniformity reflect a well-optimized formulation, ensuring reliable drug delivery performance. In vitro studies demonstrated high biocompatibility, with no significant cytotoxicity observed across various concentrations, supporting their safety for potential therapeutic applications. Pharmacokinetic analysis revealed that SRLC NPs achieved Cmax at approximately 22 h post injection, followed by sustained systemic exposure, which may contribute to prolonged GLP-1 receptor activation and metabolic regulation. Furthermore, in vivo experiments showed that the SRLC NPs effectively mitigated obesity-induced metabolic dysfunction by not only reducing excessive weight gain but also significantly improving glucose homeostasis and alleviating hepatic lipid accumulation. Histopathological analysis further confirmed notable improvements in adipose tissue morphology, highlighting the therapeutic potential of SRLC NPs in managing metabolic disorders. These findings collectively suggest that SRLC NPs represent a promising nanotherapeutic strategy for the treatment of obesity and related metabolic diseases. Their enhanced stability, biocompatibility, and efficacy underscore their potential for clinical translation.

## Data Availability

The data that support the findings of this study are available from the corresponding author upon reasonable request.

## Abbreviations

The following abbreviations are used in this manuscript:

ACN	Acetonitrile
DLS	Dynamic light scattering
DMEM	Dulbecco’s modified Eagle’s medium
DMSO	Dimethyl sulfoxide
DPBS	Dulbecco’s phosphate-buffered saline
DW	Distilled water
EDA	Ethylenediamine
EDC	1-ethyl-3-(3-dimethyl aminopropyl) carbodiimide
eWAT	Epididymal white adipose tissue
FBS	Fetal bovine serum
H&E	Hematoxylin and eosin staining
HFD	High-fat diet
iBAT	Interscapular brown adipose tissue
MD	Molecular dynamics
NHS	N-hydroxysuccinimide
NMR	Nuclear magnetic resonance
OGTT	Oral glucose tolerance test
ORO	Oil Red O staining
PK	Pharmacokinetic
RD	Regular diet
RITC	Rhodamine B isothiocyanate
RLC	Rosuvastatin–lipid conjugate
RO	Rosuvastatin calcium
RP-HPLC	Reversed-phase high-performance liquid chromatography
sWAT	Subcutaneous white adipose tissue
SEM	Scanning electron microscopy
SRLC NPs	Semaglutide–RLC nanoparticles
TBE	2,2,2-tribromoethanol
TEM	Transmission electron microscopy
TFA	Trifluoroacetic acid
UDCA	Ursodeoxycholic acid
